# Development of a one-step analysis method for several amino acids using a microfluidic paper-based analytical device

**DOI:** 10.1038/s41598-022-07408-9

**Published:** 2022-03-02

**Authors:** Akimitsu Kugimiya, Sho Wakimoto, Jiro Kohda, Yasuhisa Nakano, Yu Takano

**Affiliations:** Department of Biomedical Information Sciences, Graduate School of Information Sciences, Hiroshima City University, 3-4-1 Ozuka-higashi, Asaminami-ku, Hiroshima, 731-3194 Japan

**Keywords:** Biotechnology, Biomarkers, Health care

## Abstract

A one-step analysis method was developed for four types of amino acids using a microfluidic paper-based analytical device fabricated from chromatography filtration paper and laminate films. Aminoacyl-tRNA synthetase was used to detect each amino acid. The obtained laminated paper-based analytical device (LPAD) contained four enzymatic reaction areas. Colorimetric detection was performed based on the molybdenum blue reaction. A model method for the simple, easy, and simultaneous detection of several amino acid concentrations was suggested, in contrast to the conventional methods such as HPLC or LC–MS. The method provided a selective quantification at the ranges of 3.6–100 μM for tryptophan, 10.1–100 μM for glycine, 5.9–100 μM for histidine and 5.6–100 μM for lysine with a detection limit of 1.1 μM, 3.3 μM, 1.9 μM and 1.8 μM, respectively. LPAD fabrication was considerably simple, and the subsequent detection process was easy and required a short period of time (within 15 min).

## Introduction

Paper-based analytical devices (PADs) exhibit significant features, such as their simple and easy design for the fabrication of the shape and length of the microfluidic path, unnecessary of an external power source, and cost-effectiveness. PADs have drawn intense research attention as analytical platforms^1–5^. Although high-performance liquid chromatography (HPLC) is generally used to analyze biological compounds, it is time consuming and expensive^6^. The use of PADs to analyze biological compounds has previously been reported by other research groups. For example, Huang developed a platinum-staining method for Myoglobin as the model compound, in which the surface of colloidal gold was coated with platinum nanoshells and assessed using a test strip, such as the reagent used in pregnancy tests. They succeeded in developing a sensitive quantitative detection method for Myoglobin based on high levels of catalytic activity^7^. Another example is silica gel strips which have been developed for the detection of Fe (III) ions. The silica strips were impregnated with tannin, and because of the selective binding of tannin to Fe (III), the colorimetric detection of Fe (III) could be made by visual inspection, using anthocyanin, a red dye extracted from cabbage^8–10^. As paper test strips can be fabricated at low cost, they could easily be provided in large quantities for medical checkups and drug assessments^11^.

Free amino acid contents in blood serum serve as an indicator of a disease state, such as cancers, hepatic diseases, and diabetes; hence, they are considered useful in clinical diagnostics^12,13^. We previously analyzed a novel amino acid analysis system using aminoacyl-tRNA synthetase (aaRS) as the molecular recognition element^14–19^. aaRSs exist for each of the 20 corresponding amino acids and are involved in the biosynthesis of proteins and peptides in the body. Thus, aaRS could serve as a recognition material for amino acid analysis^20–23^.

Four amino acids, tryptophan, glycine histidine, and lysine, were used as model amino acids in this study due to their diagnostic potential for several disorders and their metabolic importance. Synthesis of serotonin from tryptophan is inhibited in patients with depression; therefore, the measurement of the tryptophan levels could have a potential use for the diagnosis of this condition^24^. Glycine is a basic component of various biomolecules such as glutathione, purine derivatives, and hemoproteins. Moreover, the intake of glycine has been reported to improve sleep quality^25^. Histidinemia is an inherited disease characterized by abnormal plasma concentrations of histidine which may result in intellectual impairment^26^. Lysine, one of the essential amino acids, is associated with the improvement of the nutritional balance in the human body^27^.

In this study, a laminated PAD (LPAD) for tryptophan, glycine histidine, and lysine with an enzymatic reaction area corresponding to the detection area was fabricated. This LPAD provided the simple, easy, and simultaneous detection of several amino acids’ concentrations, in contrast to the conventional methods such as HPLC or LC–MS. Colorimetric detection was performed based on the molybdenum blue reaction. Tryptophanyl-tRNA synthetase (TrpRS; tryptophan-specific aaRS), glycyl-tRNA synthetase (GlyRS; glycine-specific aaRS), histidyl-tRNA synthetase (HisRS; histidine-specific aaRS), and lysyl-tRNA synthetase (LysRS; lysine-specific aaRS) were used for the recognition elements of each amino acid. The analytical conditions and detectable concentration range for each amino acid were determined.

## Materials and methods

In this system, aaRS first recognizes its corresponding amino acid in the presence of adenosine 5′-triphosphate (ATP), and then aminoacyl-AMP and pyrophosphate are released (Eq. 1). The resulting pyrophosphate reacts with ammonium molybdate and 2-mercaptoethanol, which are usually used for the detection and measurement of phosphate (Eq. 2)^23^. Finally, the resulting blue shade is quantified, and the amino acid concentrations are calculated^19^.

1 $${\rm{amino}}\;{\rm{acid}} + {\rm{ATP}}\;\xrightarrow{{\rm {aaRS}}}\;{\text{aminoacyl-AMP}} + {\rm{pyrophosphate}}$$

2 $${\text{pyrophosphate}} + {\text{ammonium}}\;{\text{molybdate}}/{\text{H}}_{2} {\text{SO}}_{4} \;\xrightarrow{{{\text{mercaptoethanol}}}}\;{\text{molybdenum}}\;{\text{blue}}$$

### Materials

Amino acids, magnesium chloride hexahydrate (MgCl_2_·6H_2_O), ammonium molybdate, 2-mercaptoethanol, hydrochloric acid, sulfuric acid, and sodium hydrogen carbonate were purchased from Wako Pure Chemicals (Osaka, Japan). Tris(hydroxymethyl) aminomethane and ATP were purchased from Sigma-Aldrich Japan (Tokyo, Japan). Advantec filtration paper No. 1 and No. 5B were obtained from Toyo Roshi Kaisha, Ltd. (Tokyo, Japan), and a filtration paper MN616G was obtained from Macherey-Nagal Co. Ltd. (Duren, Germany), while 75-μm-thick polyester thermal bonding pouch film was acquired from ACCO Brands Corp. (Tokyo, Japan). TrpRS, GlyRS, HisRS, and LysRS were commissioned from Ikeda Tohka Industries Co., Ltd. (Hiroshima, Japan). The chemicals were commercial reagents of the highest grade and were used without further purification.

### Fabrication of the LPAD

**Figure 1:**
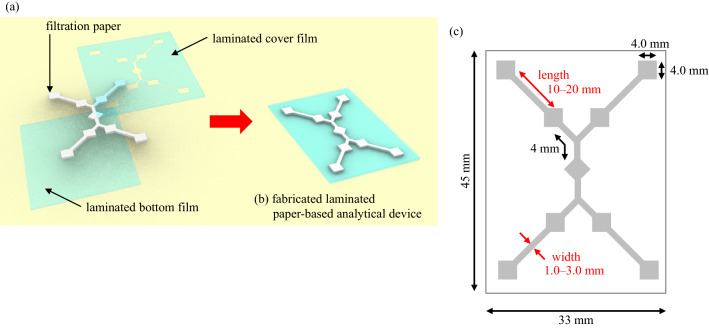
Fabrication of the laminated paper-based analytical device (LPAD). (**a**) The designed paper channels were sandwiched between the cover and bottom laminate films, aligned, and assembled. The assembly was passed through a heated laminator. The illustration was drawn with “Rhinoceros ver. 6” 3D-CAD software^28^. (**b**) An illustration of the fabricated device. (**c**) Dimensions of the microfluidic paths of the LPAD. Several path lengths and widths between the enzymatic reaction area and detection area were fabricated. The optimized length was 15 mm and width was 3.0 mm.

The LPAD was fabricated using the almost same procedure as in our previous study^19^: the LPAD pattern was designed using the controller software of a craft cutter (Graphtec CE6000-40 Cutting Plotter, Graphtec Corporation, Kanagawa, Japan). A filtration paper was affixed to an adhesive carrier sheet and cut using the cutting plotter. Paper strips were obtained after removal of the unwanted edges. The cutout pattern for the cover sheet was also fabricated. The design was exported into the craft cutter, and the cover film was cut in the same way as the paper. After cutting, the cover paper strip and the bottom sheet were aligned and assembled, as shown in Fig. 1a. The assembly was passed through a laminator heated at 100 °C (Laminator B35A3, CBC Acco Brands; Tokyo, Japan). Once the polyester films were laminated, they conformed to the outline of the paper strip. While Fig. 1b shows an illustration of the fabricated device, Fig. 1c depicts the sizes of the microfluidic paths. Several combinations of the lengths of the paths between the enzymatic reaction area and the detection area (10–20 mm) and the width of the paths (1.0–3.0 mm) were fabricated and the response was evaluated and optimized.

We determined that the optimized size was 15 mm for the length of the path between enzymatic reaction area and detection area, and the best width of the path was 3.0 mm. Advantec filtration paper No. 1 was the preferred filtration paper for the LPAD. Next, the optimized LPADs were employed for following assays.

**Figure 2:**
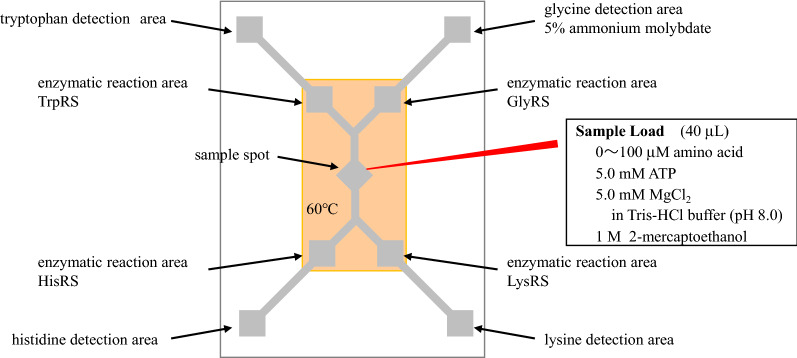
Assay protocol. Each tryptophanyl-tRNA synthetase (TrpRS), glycyl-tRNA synthetase (GlyRS), histidyl-tRNA synthetase (HisRS), and lysyl-tRNA synthetase (LysRS) was dispended to each enzymatic reaction area. Five percent of ammonium molybdate in 2.5 M sulfuric acid was dispensed to the detection area. The analyte solution was loaded onto the sample spot of the LPAD, and the spot area and the enzymatic reaction areas were heated at 60 °C using an aluminum heating block.

To prepare the enzymatic reaction area on the LPAD, TrpRS, GlyRS, HisRS, and LysRS at a concentration of 50 μM (1.0 μL) were dispended (Fig. 2). In the detection areas for amino acids on the LPAD, each solution of 5% ammonium molybdate in 2.5 M sulfuric acid (0.5 μL) was dispensed and allowed to dry at 25 °C for 30 min. The device was stored overnight at 4 °C.

### Assay protocol

The analyte solutions were prepared by dissolving each amino acid solution at various concentrations (0–100 μM). Subsequently, each amino acid solution was combined with a freshly prepared solution of ATP (5.0 mM), magnesium chloride (MgCl_2_) (5.0 mM), and 2-mercaptoethanol (1.0 M) in Tris-hydrogen chloride buffer (pH 8.0, 100 mM, total volume = 40 μL) (Fig. 2).

The analyte solution (40 µL) was loaded onto the sample spot of the LPAD. The spot area and enzymatic reaction areas were heated up to 60 °C using an aluminum heating block. After incubation for 15 min to ensure sufficient time for interaction with the pyrophosphate and color reagents, the solution was allowed to flow through the paper strips. To quantify the results, images of the paper devices were acquired using an image scanner (ES-H7200; Seiko Epson Corporation, Suwa, Nagano, Japan). The 600 dpi (24 bit) color images were analyzed using ImageJ software version 1.49^29^, and the attached command was used to equalize the color of the background to the paper strip. Images were saved, and the colors of their detection areas were inverted using the GNU Image Manipulation Program to convert the blue strength on the LPAD into brightness. The brightness and detection areas were then measured using ImageJ software. The integration signal with an arbitrary unit is obtained by multiplication of the brightness and detection area. It was defined to evaluate the progress of the enzymatic and colorimetric reactions in Eqs. (1) and (2).

## Results and discussion

In our previously reported LPAD for histidine, the enzymatic reaction was needed to be performed outside the LPAD: the reaction mixture with HisRS, histidine, ATP, and MgCl_2_ in a microtube was heated using an aluminum heating block at 80 °C for 30 min and cooled on ice for 5 min. Subsequently, the reaction mixture was loaded onto the LPAD. The factitious step with heating of the reaction mixture and pipetting of the sample were necessary^19^. In this study, an LPAD that works consecutively for both enzymatic and colorimetric reactions in one step.

### Evaluation of sizes filtration papers for microfluidics

The enzymatic reaction occurs when the enzymatic reaction mixture penetrates the detection area; consequently, the enzymatic reaction time is important and will affect the LPAD response. The type and shape of the filtration papers used for the microfluidics were evaluated using Advantec Grade No. 1, Advantec Grade No. 5B, and MN616G. Table 1 shows the sets of lengths between the enzymatic reaction and detection areas and the width of the microfluidic paths. A length between the enzymatic reaction and detection areas of 15 mm and width of the microfluidic paths of 3.0 mm was determined to be the preferred size for the LPAD. A short length and/ or narrow width path showed no or only a scarce response as the reaction mixture reached the detection area instantly. If the length was longer (20 mm), the loaded enzymatic reaction mixture could not reach the detection area. Therefore, the provision of sufficient aaRS enzymatic reaction times during penetration of the reaction mixture into the filtration paper is important to consider in designing LPADs. The density of the filtration paper fiber is also an important factor. The high-density filtration paper Advantec Grade No. 1 showed better performance in the point of the density of fiber because the reaction mixture penetrated gradually and this was sufficient for the enzymatic reaction times. The high-density filtration papers also sufficiently retained the enzyme and reagent solutions to enable the enzymatic reaction in the detection area, indicating that the stable fabrication of LPADs could be possible. Moreover, at the point of the colorimetric detection which was performed based on the molybdenum blue reaction, the depth of the color changed in a time dependent manner and became saturated. Evaluation of the color of the detection areas at 15 min after the deposition of the samples was preferred (data not shown).

**Table 1:** Evaluation of sizes of microfluidics and filtration papers.

Density of fiber		Advantec No. 1	Advantec No. 5B	Macherey–Nagel MN616G
		High	Low	Low
Length (mm)	Width (mm)			
10	1.0	×	×	×
15	1.0	×	×	×
10	2.0	×	×	×
15	2.0	×	×	×
10	3.0	△	×	×
15	3.0	○	△	△
20	3.0	×	×	×

A length between the enzymatic reaction and detection areas of 15 mm and width of the microfluidic paths of 3.0 mm was determined to be the preferred size for the LPAD. A short length and/ or narrow width path showed no or only a scarce response as the reaction mixture reached the detection area instantly. If the length was longer (20 mm), the loaded enzymatic reaction mixture could not reach the detection area. Moreover, the high-density filtration paper. Advantec Grade No. 1, showed better performance.

### Assay of the LPAD

**Figure 3:**
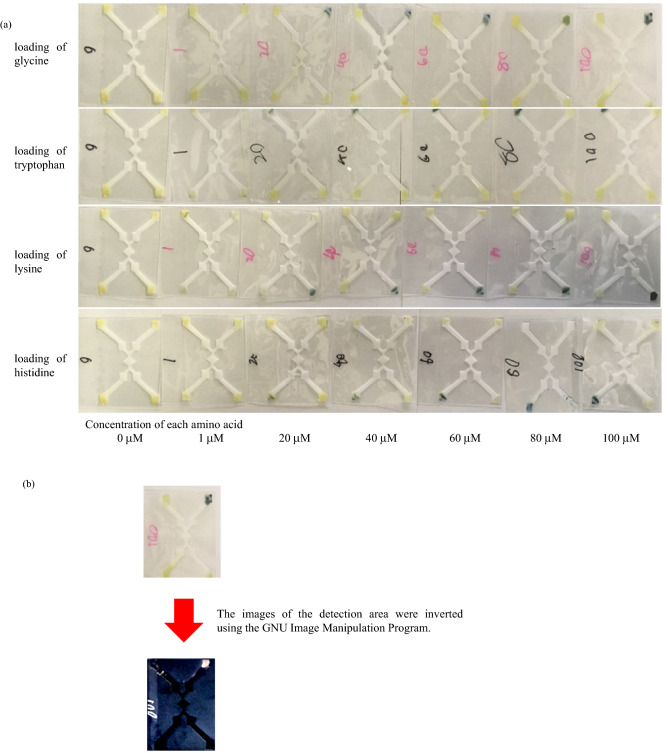
Photos of the laminated paper-based analytical devices (LPADs) after the loading of each amino acid. (**a**) Original image of each LPAD after interaction with 0–100 μM tryptophan, glycine, histidine, and lysine. Color change was observed only in the detection area of substrate amino acid. (**b**) The images were color-inverted using the GNU Image Manipulation Program.

Photos obtained after assaying the LPAD using 0–100 μM for each amino acid are shown in Fig. 3a. The color of the LPAD detection area for glycine (upper right corner of the LPAD) after loading of glycine changed from yellow to blue, whereas the detection areas for tryptophan (upper left corner), histidine (lower left corner) and lysine (lower right corner) after loading of glycine displayed no change in color. In the same manner, the color of the LPAD detection area when only the tryptophan, histidine, or lysine were loaded respectively, changed from yellow to blue, and no reactions were observed for the discordant amino acids.

The inverted images obtained using the GNU Image Manipulation Program are shown in Fig. 3b.

**Figure 4:**
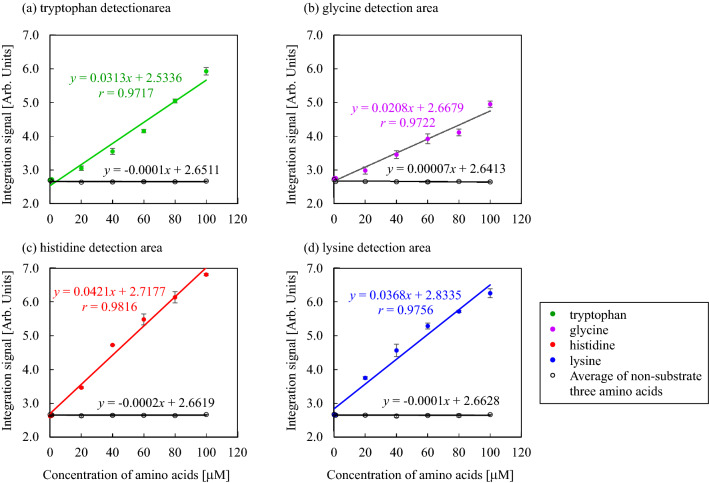
Calibration curves for tryptophan, glycine, histidine, and lysine sensing. The filled circle in each graph represents the substrate amino acid, whereas the open circles indicate the average of the integration signals of three non-substrate amino acids. Data represent the average of three measurements, and the error bars indicate standard deviations.

Figure 4 shows the calibration curves for tryptophan, glycine, histidine and lysine detection (filled circle in each graph). The horizontal axis represents the initial concentration of each amino acid, and the vertical axis represents the integration signal (arbitrary unit), which is calculated as the product of the brightness and detection area. The integration signal increased in response to the substrate amino acid addition, and good linearity ranges between 3.6 and 100 μM were obtained for tryptophan, with a detection limit of 1.1 μM (*r* = 0.9717, Fig. 4a), 10.1–100 μM for glycine, with a detection limit of 3.3 μM (*r* = 0.9722, Fig. 4b), 5.9–100 μM for histidine, with a detection limit of 1.9 μM (*r* = 0.9816, Fig. 4c), and 5.6–100 μM for lysine, with a detection limit of 1.8 μM (*r* = 0.9756, Fig. 4d).

The limit of detection (LOD) of the conventional HPLC (Hitachi Amino Acid Analyzer L-8900) is approximately 0.5 μM^6^, and slightly superior to the LOD of our LPAD. However, the measurable concentrations of each amino acid of the LPADs were within the approximate range of the amino acid levels found in the blood.

Figure 4 also shows the selectivity of the LPAD. The open circles in each graph represent the average of the integration signal of three non-substrate amino acids; the open circle in Fig. 4a (tryptophan detection area) indicates the average of the integration signal of histidine, lysine, and glycine. Each calibration curve was non-leaning, and the values were almost the same as those for 0 μM substrate amino acid; therefore, no response was observed for non-substrate amino acids. Owing to the substrate specificities of TrpRS, GlyRS, HisRS, and LysRS, these enzymes specifically bind to their corresponding substrate amino acids. Hence, the LPAD could selectively analyze the amino acids. In our previous paper, no interference was observed in the binding of the substrate amino acid to aaRS. The binding activity of aaRS to the solo substrate amino acid and the 20 amino acid mixture was almost same value; therefore, the existence of another 19 amino acids in the reaction mixture would not interfere the binding of the substrate amino acid to aaRS^14^.

### Validation of the LPAD

The reproducibility of LPAD responses to 100 μM of each amino acid among three different fabrication (3 days) and assay dates were evaluated (Table 2). Each entry was repeated three times. The coefficient of variation [CV (%)] was approximately less than 2%, and the CV values were low. These findings suggest that fabrication of the LPADs, including the cutting of the filtration papers and films, as well as the coating of the reagents, can be reproduced precisely and consistently. The LPADs showed sufficient reproducibility for each amino acid. Furthermore, as described above, they required only several micromoles of each amino acid to function, and this is consistent with the levels of amino acids in the blood.

**Table 2:** Reproducibility of laminated paper-based analytical device (LPAD) responses to each amino acid among three different fabrication and assay dates.

Amino acid	Integration signal	SD	CV (%)
Histidine	6.817	0.030	0.44
Lysine	6.264	0.132	2.11
Tryptophan	5.929	0.110	1.86
Glycine	4.952	0.091	1.84

*SD* Standard deviation, *CV* Coefficient of variation; *n* = 3 for each entry.

## Conclusion

One-step analysis device, LPAD, was fabricated for tryptophan, glycine, histidine, and lysine, using chromatography filtration papers and laminate films. In fabricating LPADs, we found that the lengths between the enzymatic reaction and detection areas and the width of the microfluidic paths were important to ensure sufficient enzymatic reaction times. High density filtration paper functioned as an effective sensor, enabling sufficient enzymatic reaction times by retaining the enzymes and reagents at the enzymatic reaction and detection areas.

The fabrication method was simple and only involved the craft-cutting of two materials at a very low cost of approximately US$2. Therefore, the LPADs could easily be produced in large quantities for medical checkups and drug assessments in the future^11^.

Tryptophan, glycine, histidine, and lysine, at concentrations ranging from several micromolars to 100 μM could be detected selectively by the colorimetric responses. The suggested LPAD in this study showed relatively good LODs in comparison with those obtained through conventional HPLC methods, and the measurable concentrations of each amino acid of the LPADs were within the approximate range of amino acid levels found in the blood. Moreover, the analysis time using the LPAD was only 15 min, while the HPLC method requires approximately 150 min for one analysis. In future studies, we plan to examine whether the assay can be used for actual samples of blood or serum.

## Abbreviations

LPAD: Laminated paper-based analytical device
PAD: Paper-based analytical device
aaRS: Aminoacyl-tRNA synthetase
TrpRS: Tryptophanyl-tRNA synthetase
GlyRS: Glycyl-tRNA synthetase
HisRS: Histidyl-tRNA synthetase
LysRS: Lysyl-tRNA synthetase
